# Serious Bacterial Infections and Hepatitis C Virus Among People Who Inject Drugs: A Syndemic or Intertwined Epidemics?

**DOI:** 10.3390/tropicalmed10010017

**Published:** 2025-01-09

**Authors:** Thomas J. Stopka, Robin M. Nance, L. Sarah Mixson, Hunter Spencer, Judith I. Tsui, Judith M. Leahy, Mai T. Pho, Jean DeJace, Judith Feinberg, April M. Young, Wei-Teng Yang, Amelia Baltes, Eric Romo, Randall T. Brown, Kerry Nolte, William C. Miller, William A. Zule, Wiley D. Jenkins, Joseph A. Delaney, Peter D. Friedmann

**Affiliations:** 1 Department of Public Health and Community Medicine, Tufts University School of Medicine, Boston, MA 02111, USA; 2 Department of Medicine, University of Washington School of Medicine, 1959 NE Pacific Street, Seattle, WA 98195, USA; rmnance@uw.edu (R.M.N.); lsmixson@uw.edu (L.S.M.); tsuij@uw.edu (J.I.T.); jadelaney@uw.edu (J.A.D.); 3 Department of Medicine, Oregon Health & Science University, Portland, OR 97239, USA; spencerh@ohsu.edu; 4 SOR Harm Reduction and Public Health Strategist, Behavioral Health Division, Oregon Health Authority, Portland, OR 97232, USA; judith.m.leahy@oha.oregon.gov; 5 Department of Medicine, Section of Infectious Diseases & Global Health, University of Chicago, Chicago, IL 60637, USA; mpho@bsd.uchicago.edu; 6 Division of Infectious Disease, Department of Medicine, University of Vermont, Burlington, VT 05401, USA; 7 Departments of Behavioral Medicine and Psychiatry & Medicine/Infectious Diseases, West Virginia University School of Medicine, Morgantown, WV 26505, USA; judith.feinberg@hsc.wvu.edu; 8 Department of Epidemiology and Environmental Health, University of Kentucky College of Public Health, Lexington, KY 40536, USA; april.young@uky.edu; 9 Division of Infectious Diseases, Perelman School of Medicine, Center for Addiction Medicine and Policy, University of Pennsylvania, Philadelphia, PA 19104, USA; wei-teng.yang@pennmedicine.upenn.edu; 10 Department of Family Medicine and Community Health, University of Wisconsin School of Medicine and Public Health, Madison, WI 53705, USA; amelia.baltes@fammed.wisc.edu (A.B.); randall.brown@fammed.wisc.edu (R.T.B.); 11 Department of Family Medicine, Boston Medical Center, Boston, MA 02118, USA; eric.romo@bmc.org; 12 Department of Nursing, University of New Hampshire, Durham, NH 03824, USA; kerry.nolte@unh.edu; 13 Department of Epidemiology, Gillings School of Global Public Health, Chapel Hill, NC 27514, USA; bill_miller@unc.edu; 14 Division of Behavioral Health and Criminal Justice Research, RTI International, Research Triangle Park, NC 27709, USA; bill.zule@yahoo.com; 15 Department of Public Health Sciences, Clemson University, Clemson, SC 29634, USA; wileyj@clemson.edu; 16 Office of Research and Department of Healthcare Delivery & Population Sciences, University of Massachusetts Chan Medical School—Baystate and Baystate Health, Springfield, MA 01107, USA; peter.friedmann@baystatehealth.org

**Keywords:** serious bacterial infections, hepatitis C virus, rural, people who inject drugs, syndemic

## Abstract

Limited research has examined the possible synergistic interrelationships between serious bacterial infections (SBIs) of the heart (i.e., endocarditis), bone, spine, brain, or joints (e.g., osteomylelitis) and hepatitis C virus (HCV) infections. We examined whether syndemic interactions existed between SBI, HCV, and substance-use-related factors in rural communities, hypothesizing that injection-mediated risks elevated the likelihood for both SBIs and HCV infections, which could be exacerbated by synergistic biological–biological or biological and social interactions. We calculated the prevalence ratios (PRs) of past-year SBI associated with each risk factor in separate models. Effect modification among significant risk factors was assessed using multiplicative interaction. Among 1936 participants, 57% were male and 85% White, with a mean age of 36 years. Eighty-nine participants (5%) reported hospitalization for an SBI in the year prior to the survey. More than half tested HCV-antibody-positive (58%); 62 (5.6%) of the participants with a positive HCV antibody result reported past-year hospitalization with an SBI. Injection behaviors were correlated with other SBI risk factors, including multiple injections in the same injection event (MIPIE), injection equipment sharing, and fentanyl use. In adjusted models, MIPIE (PR: 1.79; 95% confidence interval [CI]: 1.03, 3.11) and fentanyl use (PR: 1.68; 95% CI: 1.04, 2.73) were significantly associated with past-year SBI. Our analyses pointed to co-occurring epidemics of SBI and HCV, related to the cumulative health effects of fentanyl use contributing to frequent injections and MIPIE. Both the SBI and HCV epidemics present public health challenges and merit tailored interventions.

## 1. Introduction

The current injection drug use crisis continues to present major challenges to public health in the US. Substance use and other health outcomes are often intertwined, producing synergistic interactions that have been characterized as syndemics [1], helping to reconceptualize how biological and social factors combine to exacerbate health outcomes [2]. Syringe-mediated syndemics, which have been described previously [3], have also begun to take on new meaning, as the ever-changing nonprescribed drug supply impacts injection frequency and practices, which can, in turn, foster new risks and synergies for infectious disease acquisition [4,5]. Moving beyond the early definitions and exploration by Singer and colleagues [1,2,6], the applications of syndemics theory have expanded, with increasing relevance for public health and clinical medicine [7,8] and expanded data and analytical approaches [8,9,10,11,12].

The increased frequency of injection drug use (IDU) has resulted in a range of serious bacterial infections (SBIs). SBIs encompass skin and soft tissue infections (SSTIs), bacteremia, osteomyelitis, septic arthritis, endocarditis, and other deep abscesses [13,14,15,16]. The increase in IDU-related infectious complications is multifactorial and may reflect the evolving drug supply, where IDU was previously uncommon [17], as well as changes in the risk environment surrounding IDU (i.e., changes in the drug market, access to harm reduction services) [18,19]. IDU-related SBIs are associated with high morbidity and mortality [20], with a more than fifty-fold increase in death in some studies [21].

Hospitalization rates and hospitalization costs associated with IDU-related SBIs are important measures of the social, economic, and public health burden of IDU [22,23,24]. They also highlight critical opportunities for substance use disorder (SUD) screening, harm reduction services, and patient engagement—all interventions that can and should happen at both the hospital and community level [25]. Recent reports of increased hospitalizations for SBIs [14,17,24,26] and population-based analyses of hospitalization trends and costs among people who inject drugs (PWID) [17,23,27] indicate an urgent need to optimize resource allocation to the clinical and public health interventions that are best suited to limiting the infectious consequences of IDU. Injection-related infections are especially a concern in rural areas, which may be affected disproportionately [28]. Among rural residents, US hospitalization rates for opioid-use-associated infective endocarditis increased from 0.28 to 3.86 per 100,000 rural residents from 2002 to 2016 [29].

For decades, HCV incidence and prevalence have been exceedingly high among PWID in the US [30,31], with injection-mediated infection occurring relatively early in substance use trajectories [32], leading to high rates of infection in younger populations [33,34,35]. Historically, HCV treatment among PWID has been poorly addressed. However, evidence that direct-acting antiviral treatment is effective for people who use drugs, as well as ethical and legal arguments, has led to a loosening of restrictions for treatment and successful treatment of active injectors [36,37,38]. The rural HCV risk environment is compounded by geographic isolation, limited access to transportation, stigma, and less frequent access to harm reduction services; these factors often contribute to high-risk injection events (e.g., syringe sharing, syringe reuse, and multiple injections per injection episode [MIPIE]) [39]. Recent research focused on drug-use-related risks and HCV across eight rural sites in the US found that, compared to people who injected only stimulants, HCV antibody positivity was more prevalent among people who injected opioids alone, injected both opioids and stimulants separately, and injected both drugs with the same syringe [40].

To date, limited prior research has examined SBIs associated with IDU in rural settings or the factors associated with a higher risk of SBIs. The few studies that have assessed SBIs in rural settings have focused on infectious endocarditis [29,41]. Even fewer studies have focused on the possible synergistic interrelationships between SBIs and HCV. Prior research suggests that chronic HCV might be an independent risk factor for bacterial infections, through pathways including reduced bacterial clearance in the setting of cirrhosis or other liver injury [42,43], but the literature is scarce. High levels of streptococcal pneumonia have been documented among people living with HCV [44]. Viral hepatitis is a leading cause of chronic liver disease and cirrhosis, which has been associated with an increased risk of SSTIs [45,46], including those with unusual pathogens [47].

Homelessness, historically considered an urban issue, is a major public health challenge among people who use drugs (PWUD) in rural regions of the US and is associated with a number of drug-related behaviors that increase the risk of acquisition of bloodborne infections [48]. While rural people who inject drugs (PWID) who experience homelessness use syringe services programs (SSPs) at similar rates as those who are housed, housing instability may present barriers to more frequent SSP use [49]. SSPs provide access to alcohol wipes and increasingly to wound care specialists, offering opportunities to improve skin hygiene and reduced risks relating to the progression from SSTIs to SBIs.

**Figure 1:**
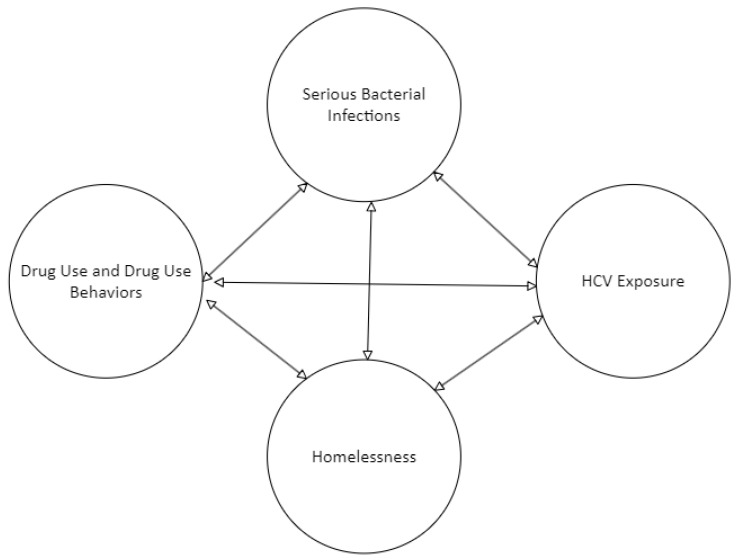
Initial hypothesis. We hypothesized that drug use (including the type of drug injected) and drug use behaviors (practicing multiple injections per injection episode [MIPIE] or injection equipment sharing), serious bacterial infection (SBI), hepatitis C virus infection (HCV), and homelessness existed in a “mutually causal” relationship [9]. Two-sided arrows represent expected relationships and potential interactions between outcomes.

Given these initial associations and potential interactions between SBIs and HCV, we sought to (1) examine associations between SBIs and HCV in a large rural sample of PWID, while considering other social factors associated with the two infections and (2) determine whether syndemic interactions (i.e., biological–biological and biological–social) existed between SBI, HCV, and other substance-use-related factors in rural communities. We hypothesized that the four outcomes of interest (drug use, HCV, SBI, and homelessness) were co-occurring and mutually reinforcing, and thus, a syndemic according to the definition given by Singer et al. [1] We operationalized “injection drug use” as the type of substance injected (fentanyl and/or methamphetamine) and IDU-related risk behaviors, including MIPIE and injection equipment sharing. This conceptual hypothesis implies that models predicting any of the four outcomes would find significant interactions between each of the other outcomes (Figure 1).

## 2. Materials and Methods

This study relied on data from seven rural sites that comprised the Rural Opioid Initiative (ROI) consortium across the United States [50]. ROI sites used modified respondent-driven sampling to recruit people reporting their past 30-day use of opioids or injected drugs [51]. Baseline survey and laboratory data were collected between January 2018 and March 2020.

The ROI study locations ranged from Northern New England (Massachusetts, New Hampshire, Vermont) to Appalachia (Kentucky, North Carolina, Ohio, West Virginia), the Midwest (Illinois, Wisconsin), and the Pacific Northwest (Oregon). All study sites were located within counties that were classified as rural as per the US Health Resources and Services Administration definition (https://www.ruralhealthinfo.org/am-i-rural, accessed on 15 August 2024).

We collected survey data from each participant at a baseline visit, which included substance use, IDU-related behaviors, date of last SBI, demographics, and housing. HCV antibody status was also measured via a rapid test or at central lab facilities. Only those participants reporting injection drug use in the past 30 days were included in this analysis.

The primary outcome of this study is a self-report of the past 12-month SBI at baseline: “Have you ever been hospitalized for a serious bacterial infection of the heart, such as endocarditis, or the bone, spine, brain or a joint, such as osteomyelitis?” “When were you last hospitalized for a serious bacterial infection of the heart, such as endocarditis, or the bone, spine, brain, or a joint, such as osteomyelitis? Don’t include times when you went to the emergency room and were not admitted to the hospital”. The six risk factors of interest were past 30-day fentanyl and/or methamphetamine use, past 30-day sharing of injection equipment, MIPIE, past 6-month homelessness, and current HCV antibody status. We focused on fentanyl and methamphetamine given that these substances were typically injected more frequently due to their short half-life and relatively short euphoric effects. We relied on HCV antibody status as a surrogate for chronic HCV infections, as confirmatory testing was not consistently employed across all sites, and our study population had low HCV treatment rates (12%) [52].

We calculated Pearson correlations between all risk factors, along with *p*-values. We ran relative risk regressions to assess the prevalence ratio (PR) of past-year SBI associated with each risk factor in separate models. We also assessed the effect measure modification between significant risk factors using multiplicative interaction. We ran the same regression models for the secondary outcome, both past-year hospitalization for SBI and HCV-antibody-positive status. We adjusted all models for age, sex, race, and site.

## 3. Results

The entire ROI cohort included 3048 participants, of whom 1936 reported past 30-day IDU. The participants were 57% male and 85% White, with a mean age of 36 years (Table 1). In the year prior to the baseline survey, 89 participants (5%) reported having been hospitalized for an SBI. All risk factors were commonly reported, ranging from 40% for fentanyl use to 81% for methamphetamine use. More than half tested HCV-antibody-positive (58%); 62 (5.6%) of the participants with a positive HCV antibody test result also reported hospitalization with an SBI in the past year.

The two injection behaviors were correlated with other risk factors (Table 2). MIPIE, injection equipment sharing, and fentanyl use were all correlated, with coefficients > 0.20. Among all participants, 40% reported current fentanyl use, and 71% reported MIPIE (Table 1), and fentanyl was positively and significantly associated with MIPIE (Table 2). Fentanyl use was negatively correlated with methamphetamine use (−0.13), although participants did not always know when they were exposed to fentanyl (Table 2). Fentanyl use, injection equipment sharing, and MIPIE were positively associated with HCV ab+ status, with fentanyl being most strongly associated with HCV (0.24). Methamphetamine use was negatively associated with HCV infection (−0.08). Finally, homelessness was not significantly associated with fentanyl, nor HCV infection (Table 2).

In adjusted models, MIPIE (PR: 1.79; 95% confidence interval [CI]: 1.03, 3.11) and fentanyl use (PR: 1.68; 95% CI 1.04, 2.73) were associated with past-year SBI (Table 3). Other risk factors had positive point estimates but did not reach statistical significance (e.g., HCV exposure was not significantly associated with SBI (PR: 1.60; 95% CI: 0.99, 2.59)). There was no significant interaction between MIPIE and fentanyl use. Homelessness was not associated with SBI and HCV. The associations were similar but slightly stronger for the secondary outcome of past-year SBI and positive HCV antibody status, MIPIE (PR: 2.40; 95% CI: 1.13, 5.10), and fentanyl use (PR: 2.35; 95% CI 1.29, 4.29).

## 4. Discussion

We explored potential syndemic interactions between SBIs, positive HCV antibody status, injection-related behaviors, and rural contextual factors across seven rural sites in the United States. Our modeling results did not support the syndemic hypothesis [1]. Importantly, the lack of significant interaction terms suggested that outcomes were not “mutually causal”. Fentanyl use and MIPIE were associated with both SBI and the combined outcome of SBI and HCV, suggesting that fentanyl use and MIPIE were contributing to the co-occurrence of these outcomes. Prior studies point to the short half-life of fentanyl leading to more frequent injection drug use [4], which could inspire MIPIE, a behavior which was documented in an earlier HIV outbreak among people who inject drugs (PWID) [5]. These findings are consistent with co-occurring epidemics in HIV and HCV infections, as well as MIPIE and oxymorphone use in Scott County, Indiana, between 2015 and 2018 [5,18]. Similarly, fentanyl use was associated with increased incidence of HCV exposure among PWID in the San Diego-Tijuana metroplex in 2020–2022 [53], as well as an HIV outbreak among PWID in northeastern Massachusetts between 2015 and 2018 [4].

Further, we did not detect a significant association between HCV exposure and SBI (*p* = 0.06), but the association trended in the direction of a potential mutually reinforcing relationship between these outcomes, meriting further research. While our modeling cannot ascribe causality, we hypothesize that fentanyl use predisposes PWID to MIPIE due to characteristics of the drug (a shorter half-life compared to other opioids) and the practice of injecting a small amount of opioids to determine the potential for fatal overdose risk due to its potency [54,55]. In sum, these findings suggest a new conceptual hypothesis of “parallel epidemics”, occurring in a step-wise rather than a mutually reinforcing manner, reflecting the cumulative health effects of one behavior (fentanyl use) contributing to another behavior (more frequent injections and MIPIE) that contributes to health outcomes [9]. Formal mediation and moderation analysis could explore this hypothesis further, given an appropriately large sample [56].

Contrary to our initial hypothesis, we also did not identify significant associations between homelessness and SBIs and HCV infections, despite high levels of homelessness and higher levels of homelessness among participants with past-year SBIs compared to those without SBIs (66% vs. 57%). We anticipated that the risk environment and milieu surrounding drug-related harms [57] among rural study participants with high levels of homelessness would foster less stability and less safe injection behaviors. While we noted modest correlations between homelessness and injection equipment sharing (Table 1), homelessness did not produce meaningful social–behavioral interactions that were associated with SBI and HCV infections in our multivariable models. In comprehensive bivariate analyses of our larger rural sample, Ballard et al. noted marginal associations between homelessness and SBIs, but significant associations between homelessness and overdose risks [48]. This is a reminder that homelessness alone may not produce the social synergistic risks that are needed to drive synergistic infectious interactions among rural PWID but may be associated with other important health outcomes. Bulled and Singer remind us that “in the case of syringe-mediated syndemics, social factors and risky syringe use as well as the nature of interactions among syringe-related infections are two primary areas of concern” [3]. The health care and harm reduction infrastructure in rural communities varies, as does the risk landscape [50]. In New England, we noted significant positive associations between geospatial access to SSPs and HCV infection risks [58], while other sites documented a wide range of social factors, including economic instability and limited economic opportunities, “as well as a lack of physically available HCV prevention and treatment services” [59]. Such varied risk landscapes could impact SBI and HCV co-infection risks and synergies in unique ways, tempering opportunities for syndemic interactions across our entire sample. However, other social factors, including transportation-related access to needed prevention and treatment services, varying health care infrastructures, and poverty levels, among other social factors, may moderate interactions and should be considered in future syndemics research. 

Our findings should be considered in light of several limitations. We analyzed the date of last reported SBI hospitalization within one year of the survey date, which implies some level of access to care. We also relied on HCV antibody status via a rapid test or at central lab facilities at the time of the survey. As a result, each infection may not have co-occurred in patients. However, the exposure to both infections likely points to a risk profile that is different from that of PWID patients who did not experience both SBIs and HCV infections. In addition, PWID participants were often recruited through harm reduction programs, which may have contributed to the recruitment of a sample with a different risk profile from the general population of PWID. We did not measure syringe reuse, across all sites, but there is evidence in some rural communities that syringe reuse is common and can be a risk factor for SBIs [60]. Finally, while our focus was on HCV and serious bacterial infections, potential syndemic relationships might exist between other infectious diseases and bacterial infections among rural PWID. HIV prevalence was very low (0.6%) in our sample, and we did not ask participants whether they had ever been infected with tuberculosis or other bacterial infections. Future syndemics-related analyses should consider exploring potential relationships and interactions among other infections.

## 5. Conclusions

Syndemics theory, since its initial inception 25 years ago [1], has expanded, with increasing relevance for public health and clinical medicine [7,8] and expanded data and analytical approaches [8,9,10,11,12]. We sought to apply syndemics theory to infectious complications related to injection-mediated risks while considering prior described syringe-mediated syndemics [3]. Through surveys and HCV testing completed as part of the Rural Opioid Initiative across several states, we found that HCV infections and SBIs co-existed in some participants. While we hypothesized that HCV and SBIs, together with the local social contextual factor of homelessness, might comprise a syndemic, our analyses instead point to parallel and intertwined epidemics of SBI and HCV, which reflect the cumulative health effects of fentanyl use contributing to frequent injections overall, as well as MIPIE. Both the SBI and HCV epidemics present serious public health and clinical challenges, indicating that prevention and treatment efforts need to be tailored to address both epidemics in concert. Future research in rural communities, informed by syndemics theory, should consider exploring additional associations and interactions among other infections, as well as a wider array of social factors that are unique to the rural risk environment.

## Figures and Tables

**Table 1:** Characteristics of rural people who injected drugs, Rural Opioid Initiative, United States, 2018–2020.

	No SBI in Past Year	SBI in Past Year	Overall
N	1847	89	1936
Age, mean (SD)	35 (10)	36 (9)	36 (10)
Male	1063 (58%)	44 (49%)	1107 (57%)
White race	1581 (86%)	73 (82%)	1654 (85%)
Site			
IL	100 (5%)	4 (4%)	104 (5%)
KY	212 (11%)	8 (9%)	220 (11%)
NC	163 (9%)	6 (7%)	169 (9%)
NE	341 (18%)	26 (29%)	367 (19%)
OH	165 (9%)	10 (11%)	175 (9%)
OR	127 (7%)	4 (4%)	131 (7%)
WI	739 (40%)	31 (35%)	770 (40%)
HCV-antibody-positive	1055 (57%)	62 (70%)	1117 (58%)
MIPIE	1305 (71%)	73 (82%)	1378 (71%)
Injection equipment sharing	1270 (69%)	69 (78%)	1339 (69%)
Homeless	1052 (57%)	59 (66%)	1111 (57%)
Current fentanyl use	725 (39%)	49 (55%)	774 (40%)
Current methamphetamine use	1503 (81%)	71 (80%)	1574 (81%)

Definitions: Serious bacterial infection (SBI); standard deviation (SD); multiple injections in one injection event (MIPIE); injection equipment sharing: past 30-day distributive and receptive sharing of injection equipment; current fentanyl use: past 30-day fentanyl use; current methamphetamine use: past 30-day methamphetamine use; homeless: past 6-month homelessness; Illinois (IL); Kentucky (KY); North Carolina (NC); New England (NE); Ohio (OH); Oregon (OR); Wisconsin (WI).

**Table 2:** Correlation between exposures of interest among rural people who inject drugs, Rural Opioid Initiative, United States, 2018–2020.

	HCV ab+ **	MIPIE	Injection Equipment Sharing	Homeless	Fentanyl
MIPIE	**0.18**				
Injection equipment sharing	**0.17**	**0.32**			
Homeless	−0.02	0.04	**0.07**		
Fentanyl use	**0.24**	**0.24**	**0.21**	0.03	
Methamphetamine use	**−0.08**	−0.02	**0.07**	**0.07**	**−0.13**

Bold entries are significant at *p* < 0.05. ** HCV ab+ infections represent HCV-positive antibody test results at the time of the study survey. Definitions: Multiple injections in one injection event (MIPIE); hepatitis C virus (HCV); antibody (ab); injection equipment sharing: past 30-day distributive and receptive sharing of injection equipment; fentanyl use: past 30-day fentanyl use; methamphetamine use: past 30-day methamphetamine use; homeless: past 6-month homelessness.

**Table 3:** Multivariable prevalence ratios for serious bacterial infection (SBI) and overlapping SBI and hepatitis C virus (HCV) infections, adjusted for age, sex, race, and study, Rural Opioid Initiative, United States, 2018–2020.

SBI Past-Year Outcome	PR	95% CI	*p*-Value
HCV ab+	1.60	0.99, 2.59	0.06
**MIPIE**	**1.79**	**1.03, 3.11**	**0.04**
Injection equipment sharing	1.48	0.89, 2.46	0.1
Homeless	1.46	0.93, 2.28	0.1
**Current fentanyl use**	**1.68**	**1.04, 2.73**	**0.04**
Current meth use	1.43	0.78, 2.62	0.3
MIPIE × Current fentanyl	0.75	0.24, 2.29	0.6
SBI and HCV Infections **	PR	95% CI	*p*-value
**MIPIE**	**2.40**	**1.13, 5.10**	**0.02**
Injection equipment sharing	1.53	0.82, 2.85	0.2
Homeless	1.50	0.88, 2.57	0.1
**Current fentanyl use**	**2.35**	**1.29, 4.29**	**0.005**
Current methamphetamine use	1.10	0.56, 2.16	0.8
MIPIE × Current fentanyl	1.28	0.27, 6.03	0.8

Bold entries are significant at *p* < 0.05. ** SBIs represented the last SBI reported in the past year. HCV infections represented HCV-positive antibody test results at the time of the study survey. Definitions: Multiple injections in one injection event (MIPIE); hepatitis C virus (HCV); serious bacterial infection (SBI); antibody (ab); injection equipment sharing: distributive and receptive sharing of syringes and other injection equipment; prevalence ratio (PR); confidence interval (CI).

## Data Availability

We welcome collaboration and encourage mentorship and the use of the ROI data, stripped of all protected health information (PHI), to enable early investigators to address meaningful questions with support to help ensure their success. Additional information can be obtained at the ROI website (ruralopioidinitiative.org) or by contacting the ROI DCC at ruralopioidinitiative@uw.edu. Follow the Rural Opioid Initiative on X @ruralopioids.
